# Postoperative complications after reconstructive surgery for cloacal malformations: a systematic review

**DOI:** 10.1007/s10151-015-1265-x

**Published:** 2015-02-22

**Authors:** H. P. Versteegh, J. R. Sutcliffe, C. E. J. Sloots, R. M. H. Wijnen, I. de Blaauw

**Affiliations:** 1 Department of Pediatric Surgery, Erasmus MC – Sophia Children’s Hospital, PO Box 2060, 3000 CB Rotterdam, The Netherlands; 2 Department of Paediatric Surgery, Leeds General Infirmary, Leeds, UK; 3 Department of Pediatric Surgery, Radboud UMC – Amalia Children’s Hospital, Nijmegen, The Netherlands

**Keywords:** Cloacal malformation, Postoperative complication, Surgery, Systematic review

## Abstract

The repair of cloacal malformations is most often performed using a posterior sagittal anorecto-vagino-urethroplasty (PSARVUP) or total urogenital mobilization (TUM) with or without laparotomy. The aim of this study was to systematically review the frequency and type of postoperative complication seen after cloacal repair as reported in the literature. A systematic literature search was conducted according to preferred reporting items for systematic reviews and meta-analyses guidelines (PRISMA). Eight records were eligible for this study which were qualitatively analyzed according to the Rangel score. Overall complication rates reported in included studies ranged from 0 to 57 %. After meta-analysis of data, postoperative complications were seen in 99 of 327 patients (30 %). The most common reported complications were recurrent or persistent fistula (*n* = 29, 10 %) and rectal prolapse (*n* = 27, 10 %). In the PSARVUP group, the complication rate was 40 % and in the TUM group 30 % (*p* = 0.205). This systematic review shows that postoperative complications after cloacal repair are seen in 30 % of the patients. The complication rates after PSARVUP and TUM were not significantly different. Standardization in reporting of surgical complications would inform further development of surgical approaches. Other techniques aiming to lower postoperative complication rates may also deserve consideration.

## Introduction

Patients with a congenital cloacal malformation undergo complex reconstruction of the rectourogenital tracts. The current surgical approach for cloacal repair was derived from the posterior sagittal anorectoplasty (PSARP), described by Peña and De Vries [1, 2]. This posterior sagittal anorecto-vagino-urethroplasty (PSARVUP) extended the anorectoplasty with a meticulous dissection of the combined vaginal–urethral walls, followed by the reconstruction of distal parts of both structures [3]. In 1997, total urogenital mobilization (TUM) was presented by Peña as a new, faster, surgical approach for certain cases of cloacal repair with better cosmetic results [4]. In TUM, the urogenital sinus is not divided into vaginal and urethral components, but mobilized *en bloc* to reach the perineum. Before the introduction of these techniques, treatment prioritized anorectal sphincter reconstruction, yet in this period, fecal incontinence was the main long-term postoperative problem [5]. Using posterior sagittal approaches, with or without the TUM, there was considerably less incontinence in the long term, but constipation or obstructive defecation became an increasingly serious problem [6].

One factor that can negatively influence final functional outcome in patients with cloacal malformations is the need for reoperations due to postoperative complications [7]. Not only is the first chance most often the best chance to deliver a good outcome, but also each trip to the operating theater carries a significant burden, both physical, psychological, and potentially financial on the patient and her carers. Postoperative complications following cloacal repair have received relatively little attention. We systematically reviewed the current literature reporting postoperative complications following cloacal repair. In this study, we aimed to develop the understanding of postoperative complications in one of the most complex congenital malformations requiring surgical intervention.

## Materials and methods

For the systematic review of the literature, the preferred reporting items for systematic reviews and meta-analyses (PRISMA) statement, checklist, and flowchart were used in order to achieve the highest standard in reporting items for a systematic review and meta-analysis [8, 9].

### Search strategy

A systematic literature search was conducted on April 19, 2014, using the PubMed, EMbase, and Web-of-Science databases. Studies were searched in PubMed using the following search terms: *(cloacal malformations OR persistent cloaca) AND complications NOT exstrophy.* For the other databases, appropriate search terms were applied concerning the postoperative outcome of patients with cloacal malformations.

### Eligibility criteria

All studies that reported postoperative complications of patients with a cloacal malformation were included. No limits were set with regard to date of publication. Case reports were excluded. Studies on the subject of anorectal malformations (ARM), in general, were only included when presenting a defined group of patients with a cloacal malformation, with the results regarding postoperative complications reported separately from the other anorectal malformations. All references of the articles we found were reviewed to include any further useful studies. Different articles that presented identical or overlapping outcome of the same study population were excluded.

### Study selection

The study selection consisted of four separate processes: (1) study identification, (2) study screening, (3) study eligibility, and (4) study inclusion. All processes were conducted by two separate reviewers (HV and IdB). Disagreements between reviewers were resolved by consensus.

### Quality assessment

Quality of the articles was scored using the checklist as proposed by Rangel et al. [10]. The checklist consisted of three subscales containing 30 items in total. The three subscales were as follows: (1) potential clinical relevance, (2) quality of study methodology, and (3) quality of discussion and stated conclusions. A maximum of 45 points could be scored. Scores ranging from 0 to 15 indicated a study of poor quality, studies scoring from 16 to 30 points were considered to be of fair quality, and scores of 31 points or higher indicated a good study.

### Data extraction

Two reviewers (HV and IdB) used predefined criteria to extract the data from included publications. The predefined criteria concerned study design, population, surgical data, and details on postoperative complications.

### Statistical analysis

Data were analyzed using SPSS (version 17; SPSS, Chicago, IL, USA). Groups were compared using a Fisher’s exact test.

## Results

### Study selection

**Fig. 1:**
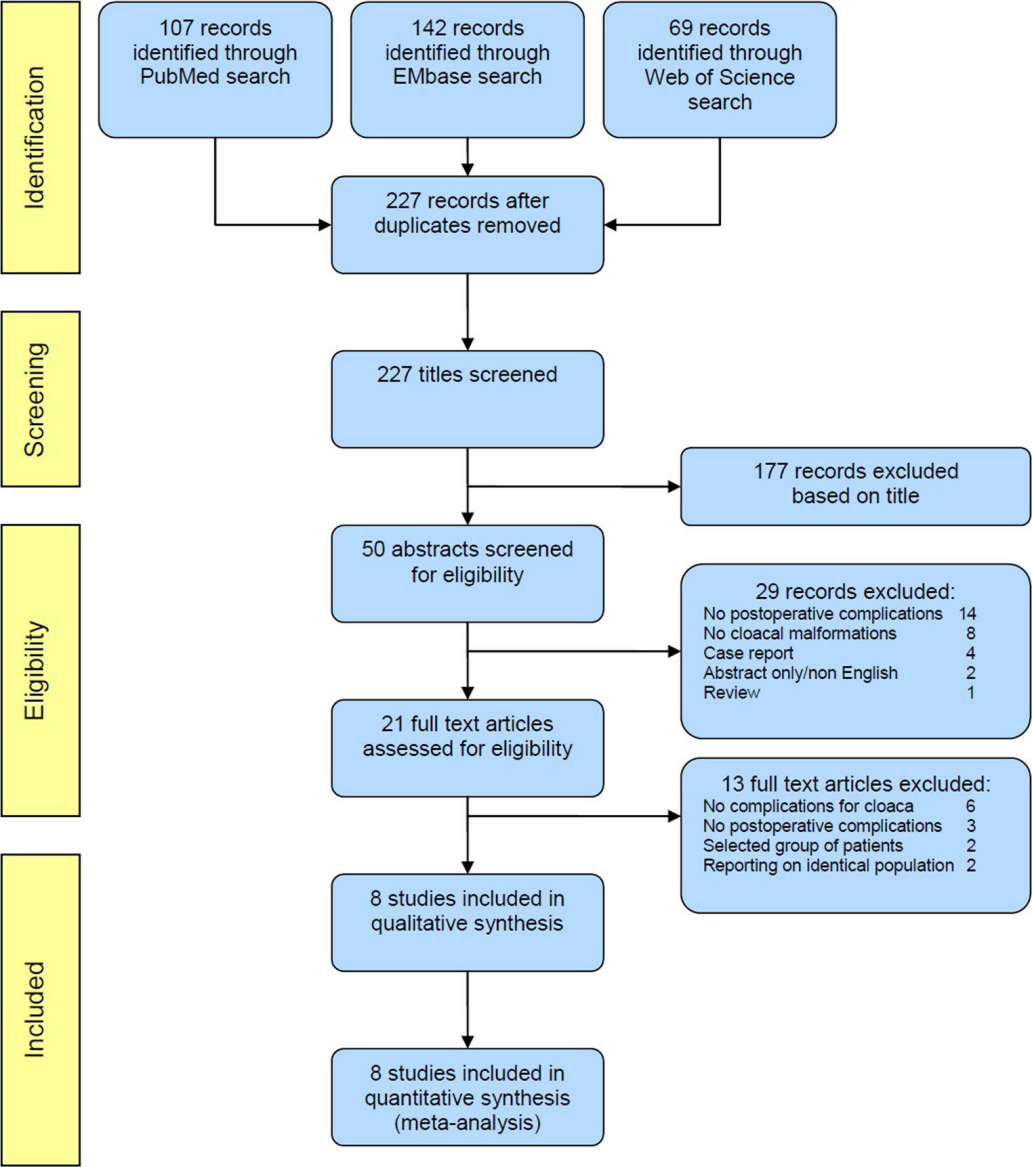
Flowchart describing systematic literature search

Adequate search terms were used for each database and resulted in 107 records (PubMed), 142 records (EMbase), and 69 records (Web of Science). After the removal of duplicates, 227 records were identified from the three databases. A total of 177 records were deemed irrelevant based on the title and excluded. Subsequently, 42 records were excluded for not meeting the inclusion criteria after assessing the abstract (*n* = 29) or the full text (*n* = 13, Fig. 1). Finally, eight studies met the inclusion criteria and were used for qualitative synthesis.

### Study characteristics

Seven of the eight studies were retrospective chart studies. One center conducted an observational cohort study [11] in which a laparoscopic rectal pull-through was conducted in ten consecutive patients with cloacal malformations (Table 1). Study quality according to Rangel’s score ranged from 10 to 31 points. A total of 597 patients were presented in the eight studies with a median of 10.5 patients per study (range 6–490 patients). However, in the largest study, postoperative complications were only reported in the 220 TUM patients. One study reported that postoperative complications were assessed within a period of 30 days after surgery, but the other studies did not report the time range in which the complications were assessed [12].

**Table 1:** Study characteristics

Author	Country	Journal	Year	Sample size	Type of surgery	Quality
Cho [14]	South Korea	J Korean Surg Soc	2011	9	PSARVUP	12
Julià [18]	Spain	Pediatr Surg Int	2010	6	PSARP	19
Leclair [16]	UK	J Urol	2007	22	TUM	19
Levitt [13]	USA	Semin Pediatr Surg	2010	490^a^	PSARVUP/TUM	16
Liem [11]	Vietnam	J Pediatr Surg	2012	10	LRP	16
Matsui [17]	Japan	J Urol	2009	11	TUM	20
Nakayama [15]	USA	J Pediatr Surg	1987	7	PSARVUP	10
Versteegh [12]	Netherlands	J Pediatr Surg	2014	42	PSARVUP/TUM	31

*PSARVUP* posterior sagittal anorecto-vagino-urethroplasty, *PSARP* posterior sagittal anorectoplasty, *TUM* total urogenital mobilization, *LRP* laparoscopic rectal pull-through

^a^Complications were only reported in the 220 TUM patients

### Type of surgery and postoperative complications

In two studies, both the PSARVUP and the TUM were used for cloacal reconstruction [12, 13]. Two studies reported the use of PSARVUP only [14, 15], and in two series, only TUM was used [16, 17]. In one study, patients were operated on by laparoscopic rectal pull-through, without initial urogenital reconstruction [11]. Julià et al. [18] described their series of patients with anorectal malformations, all of whom underwent reconstruction by the posterior sagittal approach. No details according to type of cloacal reconstruction used were reported.

**Fig. 2:**
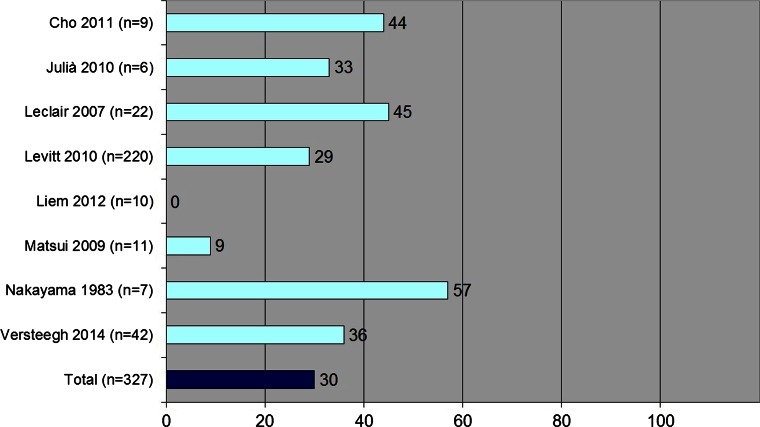
Pooled data of complications (%) reported in included studies

The reported percentages of total postoperative complications ranged from 0 to 57 % (Fig. 2). Pooled data showed that postoperative complications were seen in 99 of 327 patients (30 %). In the PSARVUP group, the complication rate was 40 % and in the TUM group 30 % (*p* = 0.205, Table 2). The most common reported complications were recurrent or persistent fistula (*n* = 29, 10 %, Table 3), rectal prolapse (*n* = 27, 10 %), and vaginal complications (such as stenosis, stricture, or occlusion, *n* = 25, 9 %). In the recurrent or persistent fistula group, 21 were urethrovaginal fistulas, four were persistent urogenital sinuses, two were rectovaginal fistulas, and one vesicovaginal fistula and one rectoperineal fistula were seen. In four of the studies, indications for reoperations were reported, with eleven of the seventeen (65 %) patients experiencing complications requiring one or more additional procedures [14, 16–18]. Nakayama et al. [15] reported that a secondary repair of their three patients with urethrovaginal fistula was being planned.

**Table 2:** Complications per type of cloacal repair

	PSARVUP^a^ (*n* = 40)		TUM^b^ (*n* = 271)		*p* value
	*n*	%	*n*	%	
Complications	16	40	81	30	0.205

*PSARVUP* posterior sagittal anorecto-vagino-urethroplasty, *TUM* total urogenital mobilization

^a^Pooled data of Cho et al., Nakayama et al., Versteegh et al.

^b^Pooled data of Leclair et al., Levitt et al., Matsui et al., Versteegh et al.

**Table 3:** Complication rates per type of complication

Study	Patients with complications^a^	Recurrent/persistent fistula or UGS	Rectal prolapse	Vaginal stricture/stenosis	Wound dehiscence	Urethral stricture/stenosis	Anal stricture/stenosis
	*n* (%)	*n* (%)	*n* (%)	*n* (%)	*n* (%)	*n* (%)	*n* (%)
Cho (*n* = 9) [14]	4 (44 %)	2 (22 %)				1 (11 %)	
Julià (*n* = 6) [18]	2 (33 %)			2 (33 %)			
Leclair (*n* = 22) [16]	10 (45 %)	4 (18 %)		3 (14 %)		2 (9 %)	5 (23 %)
Levitt (*n* = 220) [13]	63 (29 %)	13 (6 %)	26 (12 %)	18 (8 %)		6 (3 %)	
Liem (*n* = 10) [11]	0 (%)						
Matsui (*n* = 11) [17]	1 (9 %)			1 (9 %)			
Nakayama (*n* = 7) [15]	4 (57 %)	3 (43 %)		1 (14 %)	1 (14 %)		
Versteegh (*n* = 42) [12]	15 (36 %)	7 (17 %)	1 (2 %)		8 (19 %)		
Total (*n* = 327)	99 (30 %)	29 (10 %)	27 (10 %)	25 (9 %)	9 (18 %)	9 (3 %)	5 (23 %)

*UGS* urogenital sinus

^a^Some patients suffered from more than one complications

Levitt et al. [13] reported the institutional experience from a major referral center. Secondary surgery was required in 93 patients who had undergone primary surgical repair elsewhere. In this series, indications for reoperations were as follows: rectal problems (such as prolapse, stricture, retraction, dehiscence, or atresia) in 51 patients, persistent urogenital sinus in 39 patients, vaginal complications (stricture, retraction, dehiscence, atresia, or stenosis) in 34 patients, a mislocated rectum in 29 patients. Sixteen had urethrovaginal or rectovaginal fistulas, and five had urethral stricture or atresia. In addition to the recto-urethro-vaginal complications, Cho et al. [14] also reported the occurrence of bladder or urethral stones in two of their patients.

## Discussion

The surgical reconstruction of ARM has changed over the years [6]. With the introduction of the posterior approach by Peña a thorough, reproducible work-up of patients with these anomalies was established [1]. In 1997, the introduction of TUM decreased operation time and resulted in better cosmetic results [4]. Although many studies that have evaluated cloacal reconstruction have mainly focused on long-term results, this review evaluates reported postoperative complications.

Postoperative complications often require surgical treatment in this group of patients, but reoperative surgery may decrease functional outcome in patients with ARM [7]. Therefore, we assessed the number and origin of postoperative complications as a consequence of cloacal reconstruction in the current literature.

Our systematic literature search interrogated three separate literature databases with eight eligible studies subsequently found. In these studies, complication rates ranged from 0 to 57 % with a total complication rate of 30 % in 327 patients with cloacal malformations. Recurrent or persistent fistula was the most frequently reported complication occurring in 29 (10 %) of the patients in whom this was assessed.

One caveat is that complications may have been underreported; types of complications were not standardized, with each study reporting its own set of complications. Also, given the complexity of the surgical procedure, it is hard to believe reports of an absence of complications [11]. If this study would be excluded due to possibly overlooked complications, however, this would not influence the overall complication rate (31 %). The low complication rate does raise the question of whether failure to report a complication can be equated to absence of the complication for any specific study. Wound dehiscence, for example, was only reported in two studies (14–19 %) [12, 15]. It seems unlikely that there was no wound dehiscence in any of the other studies. To prevent this possible underreporting of complications, we would advocate that adequate, prospective reporting of postoperative complications in cloacal repair should at least comprise the number of each of the following: recurrent or persistent fistula or urogenital sinus, rectal prolapse, wound dehiscence, and stricture or stenosis of reconstructed structures. A recently started international prospective database on the outcome of ARM in Europe may provide useful data for this subject in the future [19].

Not all studies reported whether complications were indications for reoperations. Since the need for reoperations is likely to influence outcome, these might be of more importance than the occurrence of the complications themselves [20]. We encountered several other limitations while conducting this review; only one study reported the length of the postoperative period in which complications were assessed [12], and seven of the eight studies were retrospective. The fact that most studies comprised retrospective reports may have contributed to a possible underreporting of complications. Therefore, the complication rate for this type of complex surgery may turn out to be even higher when assessed prospectively. Furthermore, there was a wide range of study quality, with our own report as the study with the highest quality. When constructing that paper, the Rangel quality assessment score was used [10]. A high score was no more than a logical consequence of that. We advise the use of such a quality assessment score whenever constructing a retrospective report in order to achieve higher study quality. The lower scores for the other included papers, especially the paper published prior to the introduction of the Rangel scale, must therefore be seen in perspective. Also the period of time between the publication of the first study (1987) and the last study (2014) was so long that surgical practice, as well as neonatal and pediatric postoperative care, and radiological evaluation had changed. Before introduction of the posterior sagittal approach, a wide variety of techniques was used for anorectal reconstruction in ARM. All included papers, however, were reported studies conducted in the posterior sagittal era. Although surgical procedures within this time frame may have evolved a little bit further, we feel basic surgical principles have stayed the same. Therefore, we were only able to address the difference in surgical techniques, rather than non-surgical management that occurred in this period.

There were no significant differences in complication rates between the two principle techniques (40 vs. 30 %, *p* = 0.205). It is likely that there will be other differences between centers, such as in clinical experience, that will affect outcome, making comparison difficult. Another limitation of the comparison of the two surgical techniques, and thus of this study, is that the two techniques may have been used for different anatomical types of cloaca. TUM is generally used for less complex cases (with a limited length, <3 cm, of common channel) making complications less likely in this group. However, with mobilizing the urethra–vagina junction to make it reach the perineum, this technique may be prone to tension on the wound, and therefore, lead to wound dehiscence. The PSARVUP on the other hand is used for more complex cases (e.g., with a common channel >3 cm) and involves with more extensive dissection. This dissection may be a risk factor for an increased rate of complications such as recurrent fistula. It must be kept in mind that the choice of one surgical technique over the other is not as strict in clinical practice as it is in the literature. The choice is of course highly influenced by the surgeon’s experience and preference, as well as the fact that before the introduction of TUM the PSARVUP was used for all types of cloaca. This may have created a small bias in our study. However, no differences between the two techniques were observed within this review. When comparing the largest cohort in this study [13] with all the other studies, a significant difference in complication rate was not seen (29 vs. 34 %, *p* = 0.371). Of course, this center serves as a major referral center, which suggests their cases might be more complex than that of other centers.

Although TUM has been presented as an easier way to repair cloacal malformations with a shorter operation time, this approach can only be conducted in selected types of cloacal anatomy with a limited length of common channel. To our knowledge, both techniques for cloacal reconstruction have never been compared with regard to the occurrence of postoperative complications. With this systematic review including our own 25-year experience, we have demonstrated that complication rates after TUM are slightly lower than after PSARVUP, although the difference is not significant (*p* = 0.205).

With respect to postoperative complications, both PSARVUP and TUM are adequate techniques to reconstruct rectourogenital anatomy in patients with cloacal malformations, although a complication rate of 30 % could be considered to be high. Recently, laparoscopic cloacal repair has been used to perform anorectal reconstruction [11]. In the limited series presented (*n* = 10), the authors did not encounter any postoperative complications; however, a second procedure was needed for urogenital reconstruction in these patients. A lack of complications after this type of complex surgery is extremely rare, and this finding clearly needs confirmation in other studies from different centers. Depending on the capabilities of the surgeon, laparoscopic cloacal repair should be investigated as the future first-choice surgical approach. Furthermore, the field of tissue engineering, known for clinical solutions in degenerative diseases, has recently made progress in the treatment of congenital conditions [21, 22]. This novel field is developing rapidly and should be investigated in relation to improved treatment of complex congenital anomalies, such as cloacal malformations.

## Conclusions

The complex surgical reconstruction of cloacal malformations has changed over the years and is generally done by PSARVUP or TUM. This systematic review shows that postoperative complications after cloacal repair are seen in 30 % of the patients. There appeared to be no difference in complication rates between PSARVUP and TUM. The reporting of postoperative complications should be more uniform in order to determine their origin. Laparoscopic surgery and tissue engineering are matters that should be investigated as possible clinical developments in the future.
